# miR-19, miR-345, miR-519c-5p Serum Levels Predict Adverse Pathology in Prostate Cancer Patients Eligible for Active Surveillance

**DOI:** 10.1371/journal.pone.0098597

**Published:** 2014-06-03

**Authors:** Siao-Yi Wang, Stephen Shiboski, Cassandra D. Belair, Matthew R. Cooperberg, Jeffrey P. Simko, Hubert Stoppler, Janet Cowan, Peter R. Carroll, Robert Blelloch

**Affiliations:** Department of Urology, Helen Diller Family Comprehensive Cancer Center, University of California San Francisco, San Francisco, California, United States of America; IRCCS-Policlinico San Donato, Italy

## Abstract

Serum microRNAs hold great promise as easily accessible and measurable biomarkers of disease. In prostate cancer, serum miRNA signatures have been associated with the presence of disease as well as correlated with previously validated risk models. However, it is unclear whether miRNAs can provide independent prognostic information beyond current risk models. Here, we focus on a group of low-risk prostate cancer patients who were eligible for active surveillance, but chose surgery. A major criteria for the low risk category is a Gleason score of 6 or lower based on pre-surgical biopsy. However, a third of these patients are upgraded to Gleason 7 on post surgical pathological analysis. Both in a discovery and a validation cohort, we find that pre-surgical serum levels of miR-19, miR-345 and miR-519c-5p can help identify these patients independent of their pre-surgical age, PSA, stage, and percent biopsy involvement. A combination of the three miRNAs increased the area under a receiver operator characteristics curve from 0.77 to 0.94 (*p*<0.01). Also, when combined with the CAPRA risk model the miRNA signature significantly enhanced prediction of patients with Gleason 7 disease. In-situ hybridizations of matching tumors showed miR-19 upregulation in transformed versus normal-appearing tumor epithelial, but independent of tumor grade suggesting an alternative source for the increase in serum miR-19a/b levels or the release of pre-existing intracellular miR-19a/b upon progression. Together, these data show that serum miRNAs can predict relatively small steps in tumor progression improving the capacity to predict disease risk and, therefore, potentially drive clinical decisions in prostate cancer patients. It will be important to validate these findings in a larger multi-institutional study as well as with independent methodologies.

## Introduction

While prostate specific antigen (PSA)-screening has decreased PCa mortality rates in the U.S., it has done so at the cost of detecting early stage/grade PCa that might not affect a patient's life if untreated (overdiagnosis) [1]–[4]. Treating those with low risk disease results in unnecessary morbidities associated with radical interventions such as surgery or radiation (overtreatment) [5].

Active surveillance (AS) is a strategy developed to reduce the treatment of patients with low risk PCa. Such patients are monitored with physical exams, selective imaging, serial PSA assessments, and repeat biopsies. Treatment is then offered to those with signs of progression. Candidates for active surveillance protocols include those with a biopsy Gleason grade of 6 or less (no pattern 4 or 5), PSA 10 ng/ml or less, and stage T1 or T2 disease [6]. Although evidence from multiple institutions suggests that AS is a viable option for men with low-risk PCa, there are considerable misclassification rates when identifying candidates for AS [6], [7]. In one study of patients meeting criteria for AS but underwent immediate radical prostatectomy 28% were found to have a higher Gleason grade and 21% were found to have T3 disease on surgical pathology [8].

Several risk models intended to predict post-surgical pathology in low-risk patients using clinical characteristics such as age, PSA, stage, extent of tumor involvement within biopsies have been developed. However, they demonstrate an accuracy of only 61–79% complicating decision-making [9]. Due to the limitations of PSA as a surrogate for disease progression and significant legitimate concerns about biopsy sampling errors, both patients and clinicians worry that delayed treatment compromises the ability to cure disease [10]. Novel markers that reliably distinguish between low-risk versus intermediate/high-risk disease would be a solution to optimize AS protocols.

Growing evidence suggests microRNAs (miRNAs) as potentially promising markers for various malignancies [11]. These small, single-stranded, non-coding RNA molecules are involved in post-transcriptional gene regulation and have an altered expression profile in PCa tissue [12]–[14]. In addition, miRNAs detected in serum have been used to identify patients with PCa, suggesting their potential as part of a panel of serum-based markers [15], [16]. Previously, we used a microfluidic-based multiplex quantitative reverse transcriptase polymerase chain reaction (qRT-PCR) method to identify serum miRNA signatures in men with PCa that associate with the validated Cancer of the Prostate Risk Assessment (CAPRA) score for risk assessment [17]. However, this previous study did not determine whether any of these miRNAs could provide independent prognostic value. Therefore, here we aimed to identify a serum miRNA signature that could subcategorize pre-surgical patients with low-risk assessment scores into those with greater or lesser risk of progression. Due to the long follow-up required to identify patients that clinically progress and relatively short time span in which AS has been in practice, we evaluated post-surgical pathology as a surrogate marker. We found a three miRNA signature that could predict worse pathological disease than assumed based on pre-surgical biopsy. We propose that such a signature has the potential to identify a large number of patients that are unlikely to benefit from immediate surgery (i.e. low clinical score combined with an absence of a positive miRNA signature), although such a conclusion awaits a larger cross-institutional study with long term follow-up.

## Materials and Methods

### Ethics Statement

Serum and tissue samples as well as clinical data were collected between 2002 and 2012. All patients provided written consent to have their samples and linked clinical data banked for clinical and basic research purposes. The UCSF Committee on Human Research approved the consent process. For this study, all samples and data were de-identified and, therefore, did not require any additional IRB approval.

### Study Design

The study design followed the principle of prospective specimen collection and retrospective blinded evaluation (PRoBE) design [18] and reported following the REMARK guidelines [19]. All serum samples were collected routinely at the time of surgery after induction of anesthesia but prior to any procedural intervention and stored at -80°C until use. Formalin-fixed, paraffin-embedded (FFPE) radical prostatectomy tissue blocks were archived and available for analysis.

**Figure 1:**
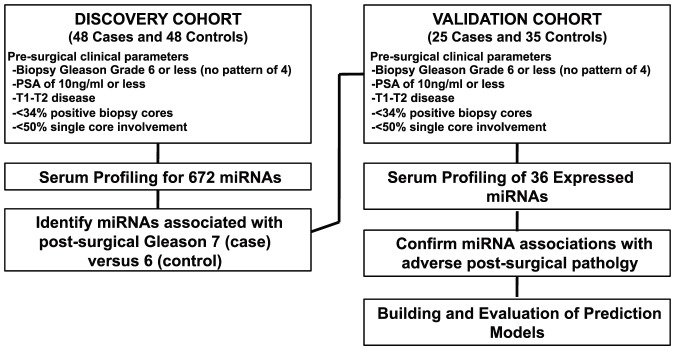
Schematic of the study design.

Patients included in the study were those opting for immediate radical prostatectomy who met the UCSF low-risk criteria for AS including biopsy Gleason 2–6 (no pattern 4 or 5), PSA less than 10 ng/ml, less than 34% of biopsy cores involved, less than 50% involvement in any single core, and clinical T1–T2 stage disease. A pathologic Gleason score of 7 of higher is associated with an increased risk of PCa mortality [20] and, therefore, was used to define adverse pathology. The discovery cohort consisted of 48 patients with a post-surgical pathologic Gleason score of 7 or higher (case group) and 48 patients with a pathologic Gleason score of 6 (control group). A validation cohort consisted of 25 cases and 35 controls. Patients were chosen from available samples maintained in a UCSF tissue bank. Patients fitting criteria above were selected from the available pool based on a random integer generator. A schematic of the study design is depicted in Figure 1.

### Microfluidics based multiplex qRT-PCR profiling of miRNAs

A microfluidic-based multiplex qRT-PCR method was used to detect miRNAs as previously described [17]. Libraries for 672 stem-loop primers, forward primers, and Taqman probes representing most known human miRNAs sequences (sequences at http://urology.ucsf.edu/blellochlab/protocols/miRNAqPCRsequences.txt) were used. Each microfluidic array evaluated the expression of 96 miRNAs for 96 samples. A total of 7 arrays were performed on the discovery cohort (672 miRNA assays). One array was performed on the validation cohort focusing only on those miRNAs detected (see below) in the discovery cohort.

Amplification plots and threshold cycle (Ct) values, a measure of the number of PCR cycles to reach a threshold line set within the exponential phase of amplification, were obtained from proprietary software on the BioMark system (Fluidigm). A higher Ct reflects lower input of measured miRNA. Ct values were confirmed through quality analysis of amplification plots. MiRNAs with a high Ct value (Ct>30) in >70% of the samples were deemed undetected and excluded from the analysis. Normalization was performed to the median Ct value of all detected miRNAs within an array for each individual patient, resulting in a ΔCT value (Tables S1–2). Normalization was performed to account for systematic variations in miRNA expression levels across individuals and arrays, so that biological differences can be more easily distinguished [21]. However, analysis of un-normalized data, controlled for amount of input serum led to analogous findings. The identity of the samples was masked until ΔCT values were obtained.

### Statistical Analysis

Initial analyses compared distributions of normalized ΔCt values from miRNAs between cases and controls in the discovery sample using graphical summaries and the Wilcoxon rank-sum test to assess significance. Logistic regression models were then fitted to candidate miRNAs singly, adjusting for age, PSA, stage, and degree of biopsy involvement including percent cores tumor positive and percent of total length of cores involved. Candidate miRNAs were included in models both as continuous measures, and as binary categorical variables. Cut-off values for the latter were determined using separate classification tree models or by the median for each miRNA. Both cut-offs gave similar results.

Prediction performance of models was distinguished using receiver operating characteristic (ROC) area under the curve (AUC) values. A separate analysis based on a random forest classification model [22] was also conducted including all detectable miRNAs as well as age, PSA, stage, and degree of biopsy involvement as predictors. The results of this analysis were used to provide an independent assessment of variable importance, and for confirmation of results from the simpler logistic models.

For the validation cohort, only those miRNAs that were detected in >30% of the samples in the discovery cohort were measured. Normalizing to all detected miRNAs run on a single array changed the breadth ofΔCt values for a given miRNA, often improving differences between cases and controls. Analytic approaches described above for discovery were similarly applied in the validation cohort. Final analyses focused on miRNAs detected as significant predictors in the discovery cohort that also were significant in the validation set. Only markers with adjusted *p*-values (based on false discovery rate methods) of at most 0.1 in discovery analyses were considered. These were modeled singly and jointly in logistic regression models also controlling for the same characteristics considered in analyses for the discovery cohort. In addition, we investigated the use of selected miRNAs to supplement the well-validated CAPRA score [23] as an index of disease risk as described in more detail in results.

Additional descriptive analyses were conducted to compare selected patient characteristics between the discovery and validation cohorts. Formal testing was based on Fisher's Exact test (for categorical characteristics) and the Wilcoxon rank-sum test (for quantitative characteristics).

All statistical analyses were performed using R (version 3.02) and Stata (version 13.1).

### In-situ hybridizations

Slides with sections (5 µm) of FFPE tumor samples from the patient cohort were deparaffinized in Xylene and hydrated through decreasing ethanol concentrations into PBS. The slides were then treated with 300 µl of proteinase-K (15 µg/ml in PK buffer, 5 mM Tris-HCl ph 7.4, 1 mM EDTA, 1 mM NaCl) at 37°c in a hybridizer (Dako) for 10 minutes. After two PBS washes, the sections were dehydrated through increasing ethanol concentrations and air-dried for 15 minutes. Double DIG-labeled locked-nucleic acid (LNA) probes (Exiqon) for individual miRNAs were denatured by heating at 90°c for 4 minutes then diluted to 40 nM using in situ hybridization buffer (Enzo Life Sciences). The sections were hybridized with 50 µl of diluted probe at 54°C overnight then washed in decreasing SSC concentrations at hybridization temperature. Following a 5 minute incubation in 0.2xSSC at room temperature, the slides were washed in PBS and blocked with 2% blocking solution (Roche) for 15 minutes. The sections were then treated with alkaline phosphatase (AP)-conjugated anti-DIG (Roche) diluted 1∶800 in blocking solution containing 2% sheep serum for 60 minutes. After two washes in AP buffer (100 mM Tris ph 9.5, 50 mM MgCl2, 100 mM NaCl, 0.1% Tween-20, 2 mM levamisol), the sections were incubated in BM Purple AP substrate (Roche) in the dark at 4°C overnight. The slides were then washed twice in PBS containing 0.1% Tween-20 and washed twice in water. Following a 30 second treatment with Nuclear-Fast Red (Vector), the slides were dehydrated though increasing ethanol concentrations then mounted. The stained slides were evaluated and graded blindly.

## Results

### MiRNA profiling in Discovery Cohort

We aimed to determine whether miRNAs from pre-surgical serum samples could predict post-surgical pathological upgrade. Clinical characteristics of the patients in our discovery cohort are summarized in Table 1. Case (post-surgical Gleason 7) and control (post-surgical Gleason 6) groups had similar distributions of race and clinical characteristics. Cases were significantly older than controls (*p* = 0.005). PSA levels also were somewhat higher in cases (*p* = 0.07). Of the 672 miRNAs tested, 36 had Ct <30 in at least 30% of the patients, which we established as a cutoff of detection (Table 2). Many miRNAs exist as clusters, where two or more miRNAs are produced from a single transcript. Nine of the 36 miRNAs are members of a single cluster, the miR17–92 cluster. MiRNAs are also grouped into families based on a common sequence at their 5′ end called the seed sequence, which largely defines their downstream targets [24]. miR-17, miR-20a, miR-92a, and miR-106a, for example, are all part of one family. Therefore, across all these PCa patients, there was an apparent enrichment for specific clusters and families of miRNAs.

**Table 1:** Patient characteristics of the discovery and validation cohorts.

DISCOVERY COHORT				
Variable		Case (n = 48)	Control (n = 48)	P-value
Age, years	Mean	60.02	56.67	0.005
	Range	45–70	43–72	
	SD	5.94	6.18	
Race	Asian/Pacific Islander	0	2	0.50
	Latino	1	0	
	Caucasian	47	46	
PSA, ng/ml	Mean	5.60	4.92	0.07
	Range	2.1–10.0	1.6–9.1	
	SD	1.74	1.85	
Clinical T-Stage	T1c	24	24	0.39
	T2	1	0	
	T2a	18	21	
	T2b	2	3	
	T2c	3	0	

VALIDATION COHORT				
Variable		Case (n = 35)	Control (n = 25)	P-value
Age, years	Mean	61.72	55.91	0.002
	Range	45–73	40–72	
	SD	6.05	7.51	
Race	Asian/Pacific Islander	1	3	0.78
	African American	0	1	
	Caucasian	23	28	
	Unknown	1	3	
PSA, ng/ml	Mean	5.72	4.67	0.04
	Range	1.73–9.70	0.64–9.99	
	SD	2.02	2.26	
Clinical T-Stage	T1c	16	20	0.75
	T2	2	2	
	T2a	3	8	
	T2b	2	4	
	T2c	2	1	

**Table 2:** MiRNAs consistently detected in serum from the discovery cohort.

MicroRNA	MicroRNA Cluster or Family
miR-17	miR-17-92 cluster
miR-19a	miR-17-92 cluster
miR-20a	miR-17-92 cluster
miR-19b	miR-17-92/106a-363 clusters
miR-92a	miR-17-92/106a-363 clusters
miR-106a	miR-106a-363 cluster
miR-93	miR-106b-25 cluster
miR-25	miR-106b-25 cluster
let-7b	let-7 cluster
miR-24	miR-181c/23b clusters
miR-939	miR-1234 cluster
miR-1234	miR-1234 cluster
miR-519c-5p	miR-1283 cluster
miR-522	miR-1283 cluster
miR-525-5p	miR-1283 cluster
miR-660	miR-188 cluster
miR-941	miR-1914 cluster
miR-1274a	miR-1274 cluster
miR-1274b	miR-1274 cluster
miR-302f	miR-302 family
miR-34c-3p	miR-34 family
miR-663	miR-663 family
miR-197	
miR-223	
miR-297	
miR-345	
miR-346	
miR-484	
miR-486	
miR-584	
miR-638	
miR-629	
miR-720	
miR-942	
miR-1208	
miR-1243	

**Figure 2:**
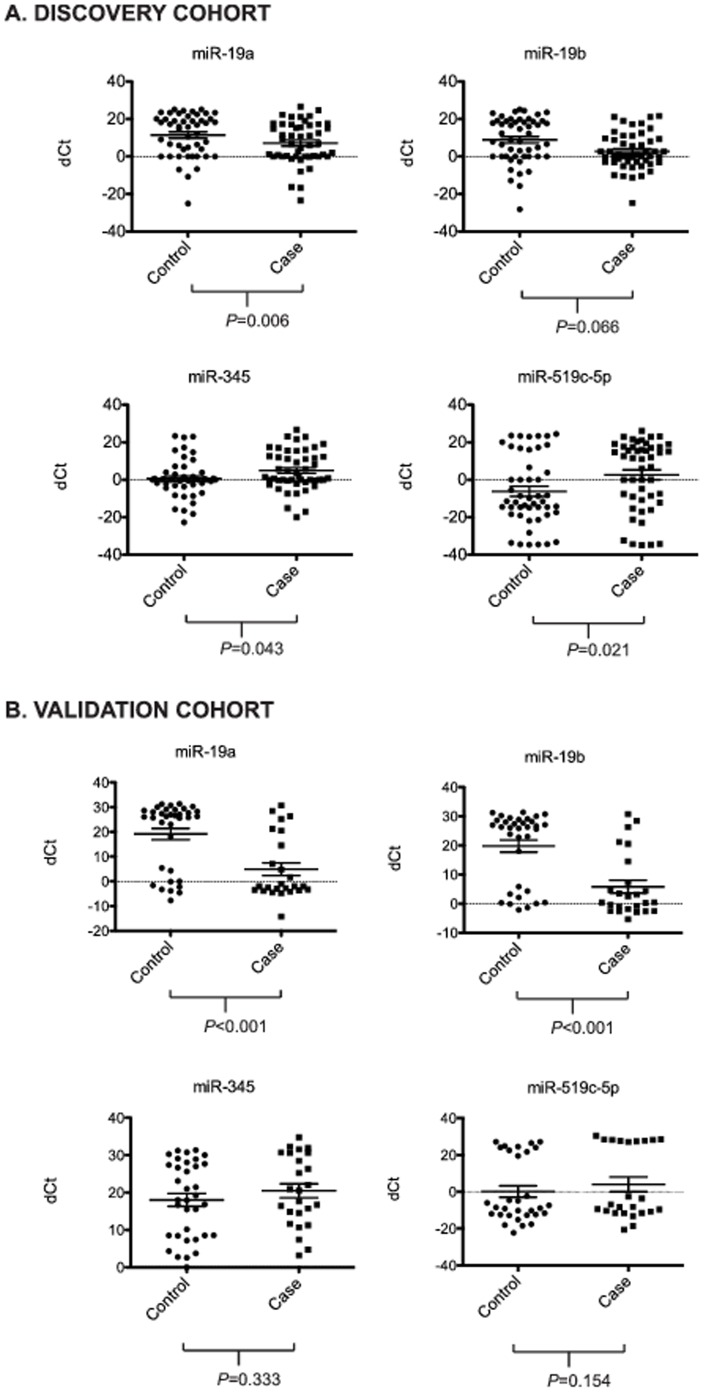
Distribution plots for serum miR-19b, miR-19a, miR-345, and miR-519c-5p delta Ct values in case versus control for a) discovery cohort, b) validation cohort. Delta Ct represents difference between Ct of individual miRNA and median Ct value among detected miRNAs within each patient. Horizontal bars represent mean +/– SEM.

**Figure 3:**
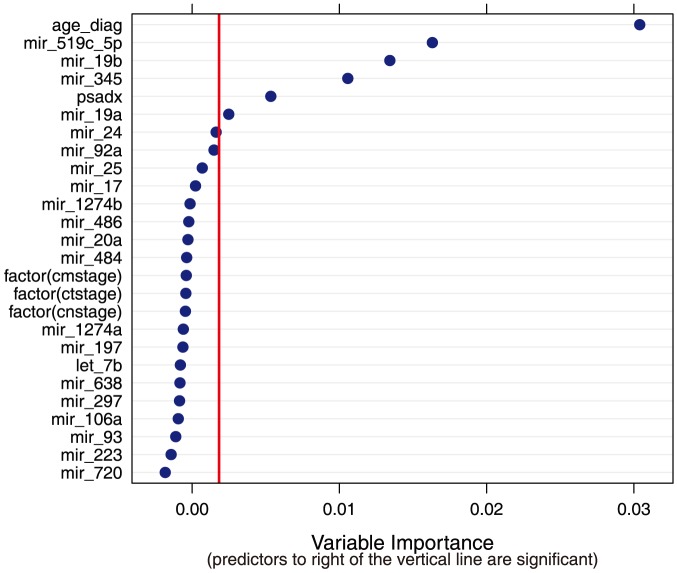
Summary of variable importance values from a random forest model fitted to the discovery dataset. Included variables are listed on the vertical axis, with corresponding variable importance for each on the horizontal axis, Importance is determined using “conditional permutation accuracy”, calculated as a the average difference in model accuracy between the fitted model and alternative versions obtained via random permutations of the variable values. Variables are considered significant predictors in the random forest if their variable importance value is above the absolute value of the lowest negative-scoring variable [43].

We next compared the serum levels of the 36 detected miRNAs between the case and control groups of the discovery cohort. Four of the detected miRNAs demonstrated differences in serum levels between the cases and controls. MiR-19a and miR-19b displayed lower ΔCt values in the case group compared to controls (Figure 2a, *p* = 0.06 and *p* = 0.07 respectively), consistent with higher serum levels in cases. Conversely, miR-345 and miR-519c-5p had significantly higher ΔCt values in the case group when compared to the controls (Figure 2a, *p* = 0.04 and *p* = 0.02 respectively), consistent with lower serum levels in the case group. Logistic regression models for predicting case/control status based on single miRNAs represented as binary indicators (with cut-off values selected using classification trees) showed all four miRNAs as highly significant predictors of outcome status (odds ratio) even when controlling for age, PSA, clinical stage, and degree of biopsy involvement (Table 3). Results were not sensitive to whether miRNAs were included in prediction models as continuous variables or as binary indicators based on median detectable levels (data not shown). A nonparametric random forest classifier including all detectable miRNAs (represented as continuous measures) as well as age, PSA, stage, and biopsy involvement ranked the following predictor variables as the six most important (in descending order): age, miR-519c-5p, miR_19b, miR-345, PSA and miR_19a (Figure 3). None of the other measured miRNAs were significantly associated with outcome status.

**Table 3:** Summaries of logistic regression models for individual MiRNAs in the discovery and validation cohorts accounting for age, PSA, stage and biopsy characteristics.

DISCOVERY COHORT (*n* = 96)				
miRNA*	OR**	95% CI (*P*)	AUC†	95% CI (*P* ‡)
miR_19a	4.82	1.20, 19.33 (0.026)	0.75	0.65, 0.85 (0.41)
miR_19b	5.57	1.81, 17.12 (0.003)	0.77	0.68, 0.87 (0.25)
miR_345	0.13	0.03, 0.47 (0.002)	0.78	0.68, 0.87 (0.19)
miR_519c_5p	0.18	0.07, 0.49 (0.001)	0.79	0.69, 0.88 (0.18)

VALIDATION COHORT (*n* = 60)				
miRNA*	OR**	95% CI (*P*)	AUC†	95% CI (*P* ‡)
miR_19a	9.22	2.27, 37.43 (0.002)	0.85	0.75, 0.95 (0.13)
miR_19b	12.67	2.72, 58.98 (0.001)	0.86	0.76, 0.96 (0.10)
miR_345	0.03	0.002, 0.37 (0.007)	0.84	0.74, 0.94 (0.09)
miR_519c_5p	0.17	0.01, 0.30 (0.034)	0.83	0.72, 0.93 (0.37)

*MiRNAs were represented in models as binary indicators, with cut-offs selected using a classification tree.

**Estimated odds ratio from a logistic regression also controlling for age, PSA, stage and degree of biopsy involvement.

† Estimated area under the ROC curve from a logistic regression also controlling for age, PSA, stage and degree of biopsy involvement.

‡ P-value comparing AUC for model including for miRNA, to model including only age, PSA, stage and degree of biopsy involvement.

### Validation of miRNA signature in independent cohort

To confirm our miRNA associations, we performed a validation study focusing only on those miRNAs that were measure in at least 30% of patients' serum samples (Table 2). The patient characteristics for the validation cohort are summarized in Table 1. Similar to the discovery cohort, there were no statistically significant differences in race, or clinical stage between cases and controls, while differences in age and PSA were significant (*p* = 0.002 and 0.04 respectively). As seen in the discovery cohort, miR-19b and miR-19a showed significantly lower ΔCt values in the case group compared to the controls (Figure 2b). Indeed, the differences were even more striking in the validation cohort (*p*<0.001 for both), likely due to normalization based on all detected miRNAs on a single array (versus spread across multiple arrays in discovery cohort). In contrast to miR-19b and miR-19a, the differences in ΔCt values for miR-345 and miR-519c-5p between the cases and controls were not statistically significant (Figure 2b, *p* = 0.333 and *p* = 0.154, respectively). However, in models adjusting for age and PSA, miR-345 and miR-519c-5p remained significant when included as binary predictors (see below). The results for analyses including miR-519c-5p were based on 55 patients (24 cases and 31 controls) due to missing values for this variable resulting from failed PCR reactions in 5 patients.

### Prognostic value of three-miRNA signature

**Figure 4:**
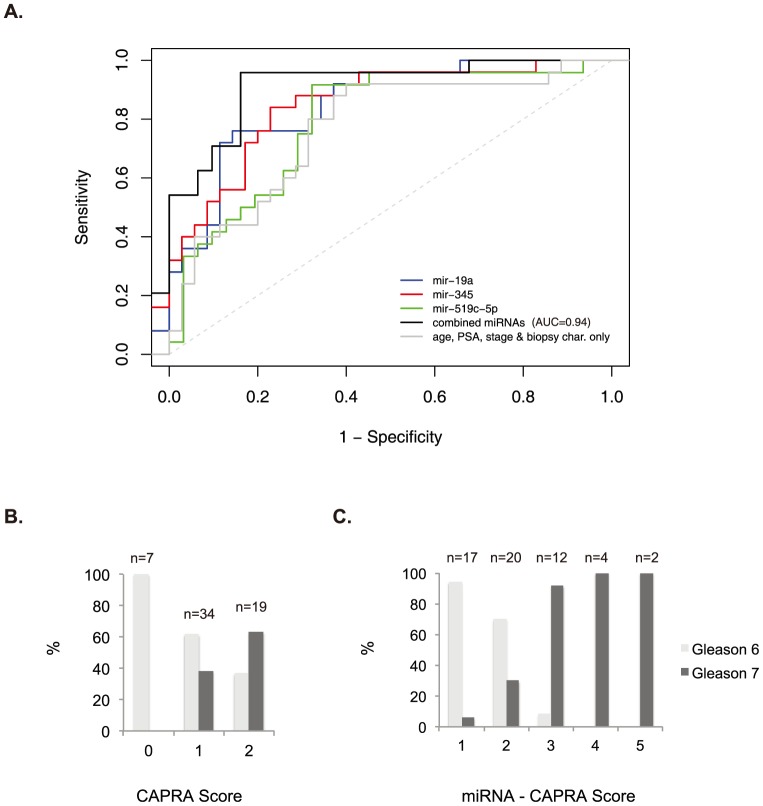
Prediction models for miRNAs. A) ROC curves for age, PSA, and stage plus/minus individual miRNAs or combination of all 3 miRNAs. B) Percentage of patients with either Gleason 6 (light grey) or Gleason 7 (dark grey) post-surgery relative to pre-surgery CAPRA score. C) Same as B, but with addition of miRNAs. A value of 1 is given for each positive miRNA and added to CAPRA score. CAPRA nomogram combines the following variables: Age at diagnosis, PSA at diagnosis, Gleason score of pre-surgical biopsy, Clinical stage, and Percent of involved biopsies.

To evaluate the ability of the confirmed miRNAs to discriminate patients with adverse pathology in the validation set, they were included in logistic regression models also adjusting for age, stage, PSA, and extent of biopsy involvement. When considered individually, all four miRNAs showed strong associations with case status, with odds ratios of 9.22, 12.67, 0.03 and 0.06 (*p* values < 0.01) (Table 3), although because of the relatively small sample size, confidence intervals for estimated odds ratios showed low precision. These miRNAs individually also showed improved discriminatory ability with AUCs of 0.85, 0.86, 0.84, and 0.83 relative to an AUC of 0.77 for a model including only age, PSA, stage, and biopsy involvement (Table 3). These improvements did not achieve significance at the 5% level (although results for miR-19b and miR-345 were significant at the 10% level). Next we evaluated models using different combinations of the miRNAs (Table 4). Notably, miR-19a and miR-19b appeared to be co-dependent, consistent with the fact that they are expressed from a common transcript, miR-17-92. Models consisting of a combination either miR-19a or miR-19b together with miR-345 and miR-519c-5p showed a AUC of 0.94, reaching high significance (*p* = 0.02 and 0.017 respectively) (Table 4 and Figure 4a).

**Table 4:** Summaries of logistic regression models for different combinations of miRNAs in validation cohort accounting for age, PSA, stage and biopsy characteristics.

Model	miRNA	OR	95% CI (*P*)	AUC	95% CI (*P*)
1	miR_19a	9.76	1.47, 64.82 (0.018)	0.930	0.86, 1.00 (0.021)
	miR_345	0.002	0.00, 0.65 (0.036)		
	miR_519c_5p	0.24	0.18, 3.21 (0.28)		
2	miR_19b	15.56	1.90, 127.22 (0.015)	0.940	0.87, 1.00 (0.017)
	miR_345	0.002	0.00, 0.42 (0.022)		
	miR_519c_5p	0.17	0.01, 3.05 (0.227)		
3	miR_19a	0.89	0.01, 55.72 (0.955)	0.940	0.87, 1.00 (0.017)
	miR_19b	17.38	0.21, 1431.62 (0.204)		
	miR_345	0.002	0.00, 0.42 (0.022)		
	miR_519c_5p	0.16	0.01, 3.14 (0.230)		

In order to identify a scoring mechanism accessible to physicians and their patients, we evaluated the utility of adding miRNA values to an established risk model, the CAPRA score [23]. Notably, our cohort had low CAPRA scores (ranging from 0–2 out of 10) as expected for AS-eligible patients. By adding one point to the score for each of the three miRNAs (miR-19a or 19b, miR-345, and miR-519c-5p) that was observed to exceed the binary cutoff value, patients could be separated into those likely to have Gleason 7—and hence intermediate risk—disease versus those that did not (Figures 4b–c). The linear trend was highly significant (*p*<0.001). Indeed, 100% of patients with a score of 4 or 5 had Gleason 7 disease post-surgically versus 7 out of 24 of patients with scores of 1 or 2.

### Origin of serum miRNAs

**Figure 5:**
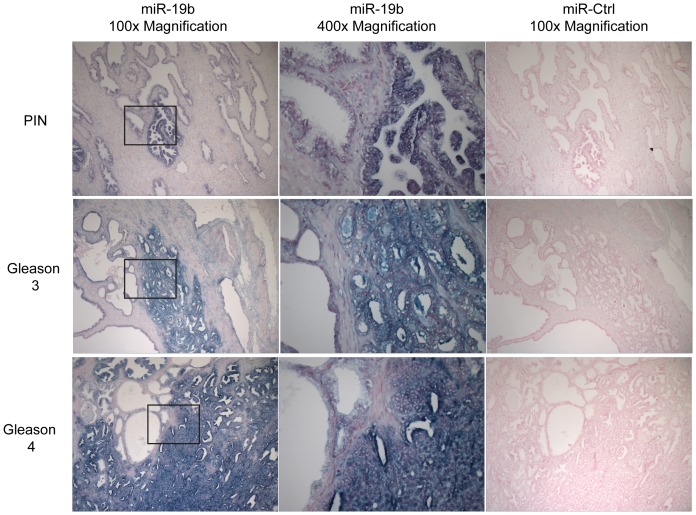
Staining for miR-19b in patient tumor samples. Images show in situ hybridization staining for miR-19b (left and center panels) and negative control, miR-295 (right panels). Top panels show area of prostate intraepithelial neoplasia (PIN). Middle panels show area of Gleason 3. Bottom panels show area of Gleason 4. Areas of PIN, Gleason 3, and Gleason 4 all stain strongly for miR-19b probe (blue staining). Note each panel has area of normal-appearing epithelium, most clearly seen on the left of each center panel. These areas show much lower staining. Right and left panels, 100× magnification. Center panels, 400× magnification. Center panels represent boxed areas in left panels.

The uncovered serum miRNAs may arise directly from the tumor or from the body's response to the tumor. In order to determine whether the predictive miRNAs are present in prostate tumors, we performed in situ hybridizations with probes for miR-19b on FFPE tumor samples from patients in our cohort (*n* = 10). We focused on miR-19b as it reached significance in both the discovery and validation sets. Note that the miR-19b probe is unlikely to discriminate between miR-19a and miR-19b due to their highly similar sequences. Staining for miR-19b was strong in areas of high-grade PIN, Gleason pattern 3, and Gleason pattern 4, but absent in normal appearing epithelium (Figure 5). However, there was no obvious difference in intensity of staining with increasing grade from PIN to Gleason pattern 4. Therefore, while miR-19 cellular levels clearly increased with transformation, it was unclear if there was any further increase within the cells with pathological progression.

## Discussion

As post-transcriptional regulators of gene expression, miRNAs act as both oncogenes and tumor suppressors [25], [26]. Aberrant miRNA signatures have been demonstrated in prostate tumor samples and have been detected in serum of men with PCa [12]–[16], [27], [28]. We previously identified a miRNA signature associated with patient risk as calculated by CAPRA scores, which predict metastases, PCa mortality, and all-cause mortality [17]. However none of these previous studies addressed whether miRNAs could provide additional information beyond what can be predicted by current parameters including PSA, imaging, and biopsy. Here, we discovered and validated four serum miRNAs, miR-19a, miR-19b, miR-345, and miR-519c-5p which in combination and when added to the current parameters, significantly improved prediction of adverse pathology within the prostate. Individually, only miR-19a/b reached significance in both the discovery and validation sets.

A predictive model including age, PSA, stage, and biopsy involvement demonstrated an AUC value of 0.77 in identifying adverse pathology in post-surgical specimens, the addition of 3 miRNAs (miR-19a or miR-19b plus miR-345 and miR-519c-5p) demonstrated an AUC value of 0.94. Furthermore, inclusion of the miRNA signature in a currently used risk score (CAPRA) improved prediction of post-surgical upgrade supporting its clinical utility. These improvements are the first demonstration to our knowledge of the independent predictive value for serum miRNAs above currently available clinicopathological measures in a highly relevant scenario. While almost 50% of newly diagnosed patients now meet the low-risk criteria for AS protocols, only 10% of such patients undergo AS [29], [30]. Having additional markers that can further segregate patients will be critical to improving these numbers.

Serum-based markers are advantageous as such tests are relatively non-invasive and can be conveniently used to monitor patients. Markers obtained from serum avoid concerns of biopsy sampling error, which is a significant limitation of tissue-based assays [31], [32]. The origins of miRNAs in human serum remain largely unclear. In situ hybridization on prostate samples from patients in our cohort demonstrated the presence of miR-19b in all areas of transformed epithelium from high-grade PIN to Gleason 4 pattern. In contrast little to no staining was seen in the stroma or normal appearing epithelium. The presence of the miR-19b within the low-grade tumor tissue, but diminished levels in corresponding serum raises the question of the source of miRNA in serum of patients with higher-grade tumors. One exciting possibility is that the miR-19b is only secreted upon progression even though it is expressed within the transformed cells early in tumor development. Alternatively, cells may need to breach structural elements within the tissue for miR-19b to reach the blood. Finally, the miRNAs may derive from the body's response to the tumor (e.g. the immune response). In summary, the cellular origins for the changes in miRNA levels in the serum with pathological progression remains to be determined.

miR-19 is a part of group of miRNAs processed from a single transcript, miR-17–92 cluster [9]. This cluster yields six mature miRNAs (miR-17, miR-18a, miR-19a, miR-20a, miR-19b, miR-92) and is located at 13q31.3, a region amplified in multiple hematopoietic and solid tumor malignancies [33]–[35]. In a B-cell lymphoma model, over-expression of *miR-17-92* with *c-myc* promotes tumor formation, demonstrating the oncogenic activity of the cluster [35]. miR-19 appears to be an essential oncogenic component of *miR-17-92* by targeting the tumor suppressor, *Pten*, resulting in activation of the Akt-mTOR pathway [36], [37]. Given the well-documented role of *Pten* in prostate cancer and that alteration is found in 30–70% of patients at the time of diagnosis, it seems highly likely that elevation of miR-19a & miR-19b seen in the tissue is functionally associated with tumor development [38]–[40].

Other serum miRNAs that differ from the ones uncovered here have been found to be elevated in prostate cancer patients. A particularly exciting example is Mir-141, which has been shown to be elevated in prostate cancer patients with advanced disease such as in those with lymph node or bone metastasis [15], [27], [28]. In our study, miR-141 was not detected in patients meeting the low-risk criteria for AS. Therefore, although miR-141 may be a potential marker for patients with more advanced PCa, it is unlikely to have value for candidates of AS deciding whether to undergo radical treatment. In addition to disease state, conflicting results among studies may also be due to variations between methods of serum miRNA profiling. Individual qRT-PCR-based protocols and microarray assays may yield different miRNA signatures. Although there is currently no gold standard for serum miRNA detection, we have previously shown that our multiplex qRT-PCR method is accurate by measuring miRNA levels in knockout systems lacking canonical or both canonical and non-canonical miRNAs [17].

In this study, adverse pathology was defined as a Gleason score of 7 on post-surgical specimen. A Gleason score of 7 is associated with an increased risk of disease-specific mortality and remains the best single predictor for behavior of disease [20]. However, a Gleason score of 7 is not a guarantee of future clinical progression. Recent studies suggest that even intermediate-risk patients with a Gleason score of 7 demonstrate excellent survival on AS with short to intermediate follow-up [41]. As more patients enroll in AS and data from ongoing trials mature, studies will need to be performed to determine whether the miRNAs predict future metastasis or disease-specific mortalities. In addition, prospective trials will be needed to determine whether they are valuable tools for monitoring patients while undergoing AS.

In conclusion, this study identifies a serum miRNA signature that can act as an independent prognostic marker in PCa. Furthermore, it shows how serum miRNAs can be used to identify relatively small steps in tumor progression allowing increasing clinical refinement of disease status. Future studies will be required to validate these markers in a large cohort across institutions as well as to associate with long-term outcomes, especially patient morbidity and mortality. Additionally, it will be important to validate these miRNAs with additional methodologies of serum quantification, which are rapidly improving providing greater sensitivity and specificity.

**Note added to revision**: While this manuscript was under review, Tilley and colleagues published data identifying miRNAs that could predicted risk for biochemical recurrence among patients with Gleason 7 or greater disease who underwent radical prostectomy providing additional evidence for the potential of miRNAs providing prognostic information beyond current clinicopathological parameters [42].

## Supporting Information

Table S1 Delta Ct values for discovery cohort.
(XLSX)

Table S2 Delta Ct values for validation cohort.
(XLSX)
